# Complete Genome Sequence of Lactobacillus reuteri WHH1689, Isolated from Traditional Chinese Highland Barley Wine

**DOI:** 10.1128/genomeA.00425-18

**Published:** 2018-06-07

**Authors:** Su Chen, Lin Chen, Lie Chen, Xueliang Ren, Hongjuan Ge, Guokai Kang, Sheng Sun, Zuoguo Chen, Yong Sun, Yanjun Li

**Affiliations:** a Key Laboratory of Food and Biological Engineering of Zhejiang Province, Research and Development Department, Hangzhou Wahaha Technology Co. Ltd., Hangzhou Wahaha Group Co. Ltd., Hangzhou, China

## Abstract

Here, we report the complete genome sequence of Lactobacillus reuteri WHH1689, which was isolated from traditional Chinese highland barley wine in the Tibetan Plateau of China. The genome consists of a circular chromosome (2.04 Mb).

## GENOME ANNOUNCEMENT

Probiotics are live microorganisms that, when administered in adequate amounts, confer a health benefit on the host (1). Most probiotic bacteria are lactic acid bacteria (LAB). Because of their long history of safe use and potential therapeutic benefits for human health, LAB have received increasing attention (2). Among the LAB, Lactobacillus is the most common genus (3). Lactobacillus reuteri WHH1689 was isolated from highland barley wine in the Shigatse area of Tibet, China (4).

Genomic DNA was extracted and purified using a DNA extraction kit for bacteria (Promega, USA) according to the manufacturer’s instructions. The genome was sequenced at the Majorbio Co., Ltd. (Shanghai, China). PacBio single-molecule real-time (SMRT) whole-genome sequencing was performed using a PacBio RS II sequencer with P6-C4 chemistry. Two SMRT cells were used for sequencing, thereby yielding 44,311 adapter-trimmed reads (subreads) with an average read length of approximately 7,467 bp, which corresponded to approximately 161-fold coverage. After filtering, the reads were assembled with the Hierarchical Genome Assembly Process (HGAP) 2.3.0 (5), resulting in one circular chromosome comprising 2,044,184 bp with a G+C content of 39.3%.

The tRNAs were predicted by tRNAscan-SE 1.3.1 (6), rRNAs were predicted by Barrnap 0.4.2 (https://github.com/tseemann/barrnap/). The coding DNA sequences (CDSs) were predicted by three software programs, Glimmer 3.02 (7), GeneMarkS 4.3 (8), and Prodigal 2.63 (9), and corrected manually. Finally, functional annotation was performed based on homology searches against the Cluster of Orthologous Groups (COG) protein database (10).

A total of 2,242 genes were identified in the genome sequence, including 69 tRNA genes and 18 complete rRNA operons. The COG annotation results revealed that approximately 69.8% of the protein-coding genes (proteins) were assigned to at least one functional category. A total of 327 CDSs were allocated to replication, recombination, and repair (category L), followed by translation, ribosomal structure, and biogenesis (J), with 136 proteins. Further comparison analysis of the genome with different strains in the same genus is now under way.

### Accession number(s).

This whole-genome sequence and the annotation data have been deposited at GenBank under the accession number CP027805.
